# Woody plant functional traits and phylogenetic signals correlate with urbanization in remnant forest patches

**DOI:** 10.1002/ece3.10366

**Published:** 2023-07-31

**Authors:** Jingyi Yang, Zijin Wang, Ying Pan, Yanjun Zheng

**Affiliations:** ^1^ College of Forestry Guizhou University Guiyang China

**Keywords:** functional traits, phylogenetic signals, species group, urban remnant forests, urbanization, woody plants

## Abstract

Exploring the alterations in functional traits of urban remnant vegetation offers a more comprehensive perspective on plant assembly within the context of urbanization. While plant functional traits are influenced by both environmental gradients and the evolutionary history of plant species, the specific mechanisms by which urbanization mediates the combination of functional traits and the evolutionary history of remnant vegetation remain unclear. To examine the relationship between functional traits and phylogenies of remnant vegetation and urbanization, we classified the woody plant species surveyed in 72 sample plots in nine remnant forest patches in Guiyang, China, into four groups (urban, rural, middle and general groups) according to their location under different levels of urbanization and measured nine functional traits of these species. The phylogenetic signals of each functional trait of the four species groups were then quantified based on Blomberg's *K*. Furthermore, we analysed the correlations between functional traits and species abundance using phylogenetic generalized least squares. The results showed that significant phylogenetic signals were detected in more functional traits of the urban group than other groups. Thirteen and three significant relationships between functional traits and species abundance were detected for tree and shrub species after removing phylogenies. Tall tree species were more abundant in the urban group, while the general group favoured the species with adaptable traits (low height and high leaf area and C/N). Overall, we demonstrate that urbanization drove shifts in plant functional traits in remnant forests after combining the phylogenetic patterns.

## Graphical abstract

The study found significant phylogenetic signals in many plant functional traits, especially for urban groups. Moreover, the phylogenies significantly affected our ability to detect dominant functional traits for different groups of woody plant species. However, most previous studies only focused on taxonomic and functional filtering while ignoring the effect of phylogenies.

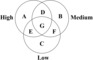

## 1 INTRODUCTION

Remnant forests within urban areas are defined as natural or semi‐natural forests that have persevered through the urbanization process and have not been subjected to clearing for urban development purposes (Zipperer, 2002). Urban remnant forests are hotspots biodiversity of cities but have been severely destroyed and threatened (Siraj et al., 2018; Yang et al., 2007). Many previous studies have proven the direct and indirect effects of urbanization on the taxonomic diversity of remnant forests (Hahs & McDonnell, 2007; Ramalho et al., 2014; Yang, Luo, et al., 2022; Zipperer, 2002). Forest remnant size has been found to have a positive correlation with plant species diversity (Malkinson et al., 2018; Stiles & Scheiner, 2010). The heterogeneity of the urban matrix has been identified as a mediator between patch sizes and the taxonomic diversity of plants in remnant forests (Yang, Luo, et al., 2022). However, taxonomic diversity is limited to the number and distribution of plant species in a given community (Ricotta et al., 2008). Studies on the process‐based mechanisms that dictate the relationship between urban environments and plant diversity within remnant forests are grossly inadequate.

Functional traits are useful tools for studying process‐based plant responses to biotic and abiotic factors in urban environments, which promote species coexistence (Knapp et al., 2009; Williams et al., 2009, 2015). Urbanization encompasses general ecological phenomena and a range of specific interfering factors (McDonnell & Pickett, 1990; Sukopp, 2004). The crucial role of functional traits in driving remnant vegetation responses to urbanization has been demonstrated in previous studies. For instance, urbanization favoured plant species with small sizes and wind‐dispersed seeds in urban remnant forests (Guerra et al., 2017). However, urbanization reduced the number of plant species with limited dispersal capacity, such as animal‐dispersed or insect‐pollinated species (Huang et al., 2013; Ramalho et al., 2018). As taxonomic diversity merely considers the number and distribution of plant species, studying changes in the functional traits of urban remnant vegetation offers a more comprehensive comprehension of the plant assembly process amidst urbanization.

However, it is worth noting that plant functional traits are influenced not only by environmental gradients but also by the evolutionary history of plant species (Ma et al., 2018). Typically, closely related species exhibit little variation in functional traits, while more distantly related species tend to demonstrate greater differences in functional traits (Felsenstein, 1985). Therefore, phylogenetic signals are usually applied to test the correlation between functional traits and phylogenies (Blomberg et al., 2003). High levels of phylogenetic signals indicate a strong correlation between the functional traits of plants and their evolutionary history (Liu et al., 2015). For instance, Yang et al. (2014) discovered that functional traits in two extensive forest dynamics plots demonstrated a remarkable level of phylogenetic signals. However, the extent of the phylogenetic signal varies between different plant traits, with more instances of a phylogenetic signal observed in structural traits as opposed to physiological traits (Avila‐Lovera et al., 2023). Not all functional traits, of course, show a significant phylogenetic signal. In cases where closely related species are exposed to heterogeneous environmental conditions, environmental factors may outweigh the role of phylogeny (Schmidt et al., 2018). Conversely, species that are distantly related on the phylogenetic tree may show convergent evolution when they reside in similar environments (Butcher et al., 2007). As such, the strength of the phylogenetic signal in functional traits can vary and is not always prominent. Urban environments are more likely to exhibit a strengthening association between functional traits and system development, due to the presence of obvious heat island effects, high levels of habitat fragmentation, severe pollution and significant human disturbance (Buyantuyev & Wu, 2010; Escobedo et al., 2011; Jellinek et al., 2004). These factors can alter species' gene flow and adaptive evolution, resulting in an increase in the number of closely related species with similar functional traits.

The participation of the entire community in various processes will be influenced not just by the existence of certain species and their traits but also by their varying abundance (Stuart‐Smith et al., 2013). The abundance of different species in a community reflects their relative importance in the community structure, and the abundance also reflects a species' ability to occupy resources and allocate resources to each individual (Mouillot et al., 2007). The greater the number of individuals in a population, the greater amount of resources they are able to consume in a community. Conversely, populations with fewer individuals have less resources available for their use within a community. Studies have demonstrated the importance of functional traits in determining the success or failure of species in certain environmental conditions (Li et al., 2021). As such, these traits are frequently employed to identify variations in performance across species and determine the extent to which such variations have adaptive significance, which can ultimately impact the relative prevalence of co‐existing species (Garnier & Navas, 2011).

Previous research has investigated the impact of urbanization on functional traits of remnant vegetation (Huang et al., 2013; Ramalho et al., 2018; Yang, Wang, et al., 2022). However, the influence of phylogenies has not been widely explored. Failure to account for the phylogenetic patterns can lead to functional trait variables being dependent on one another, which can obscure the ways in which environmental conditions filter plant species into community assembly. It has been demonstrated that several functional traits exhibit strong phylogenetic signals in natural environments (Donoghue, 2008; Li et al., 2017; Webb et al., 2002). Nonetheless, it remains uncertain whether urbanization mediates the phylogenetic signals of functional traits in remnant vegetation within human‐dominated landscapes. In instances where a phylogenetic signal exists, the correlation between plant functional traits and plant species abundance which can reflect the community structures based on functional traits (Cao et al., 2013), must account for the influence of phylogenies. Moreover, species abundance reflects the relative significance and resource utilization of a species in a community, which is also impacted by phylogenies (Mi et al., 2012). Therefore, exploring the functional and phylogenetic traits of remnant vegetation can facilitate elucidation of the mechanisms driving the relationship between urban environments and community assembly patterns (Burns & Strauss, 2012; Jin et al., 2021).

This study aims to investigate the ecological strategies of remnant vegetation based on their functional traits by removing the effects of phylogenetic nonindependence. The primary goal is to enhance our understanding of how plant species support populations in urban environments. Specifically, we will examine the variation in the functional traits and phylogenies of woody plant species in remnant forest patches at various levels of urbanization. We selected a recently rapidly urbanizing city—Guiyang, China—as a case study. The specific objectives in this study include (1) grouping the woody plant species in urban remnant forests into different species groups according to their occurrence at the sites under different levels of urbanization; (2) measuring the functional traits and phylogenetic signals of different species groups of woody plants; and (3) exploring the correlation between functional traits and abundances of different species groups after removing phylogenetic signals. In this study, we categorized the woody plant species into tree and shrub species, as previous studies have confirmed that tree and shrub species exhibit dissimilar responses to urbanization owing to their distinct sizes and regeneration rates (Yang, Wang, et al., 2022). Our expectation is to discover noteworthy correlations between functional traits and species abundances. This can be achieved by eliminating the phylogenetic signals and identifying the changes in functional traits as the abundance of different species groups increases. Such an approach would improve our understanding of how environmental conditions influence the selection of plant species in community assembly. By avoiding interdependence among the functional trait variables, we can gain more accurate insights.

## 2 METHODS

### 2.1 Study area

We conducted this research in the metropolitan area of Guiyang, China. The study area is in a subtropical zone and is famous for its karst landform. The average altitude of Guiyang is 1100 m, and the annual total precipitation is 1130 mm. The average temperature in summer ranges from 19 to 26°C, while in winter the average temperature is between 2.5 and 12°C. The city has a higher total species richness due to its complicated topography and humid climate. The subtropical evergreen broadleaf forests are the zonal vegetation type of this region. Vegetation from the Fagaceae and Lauraceae families is dominant in the study area.

### 2.2 Field survey and data collection

**FIGURE 1:**
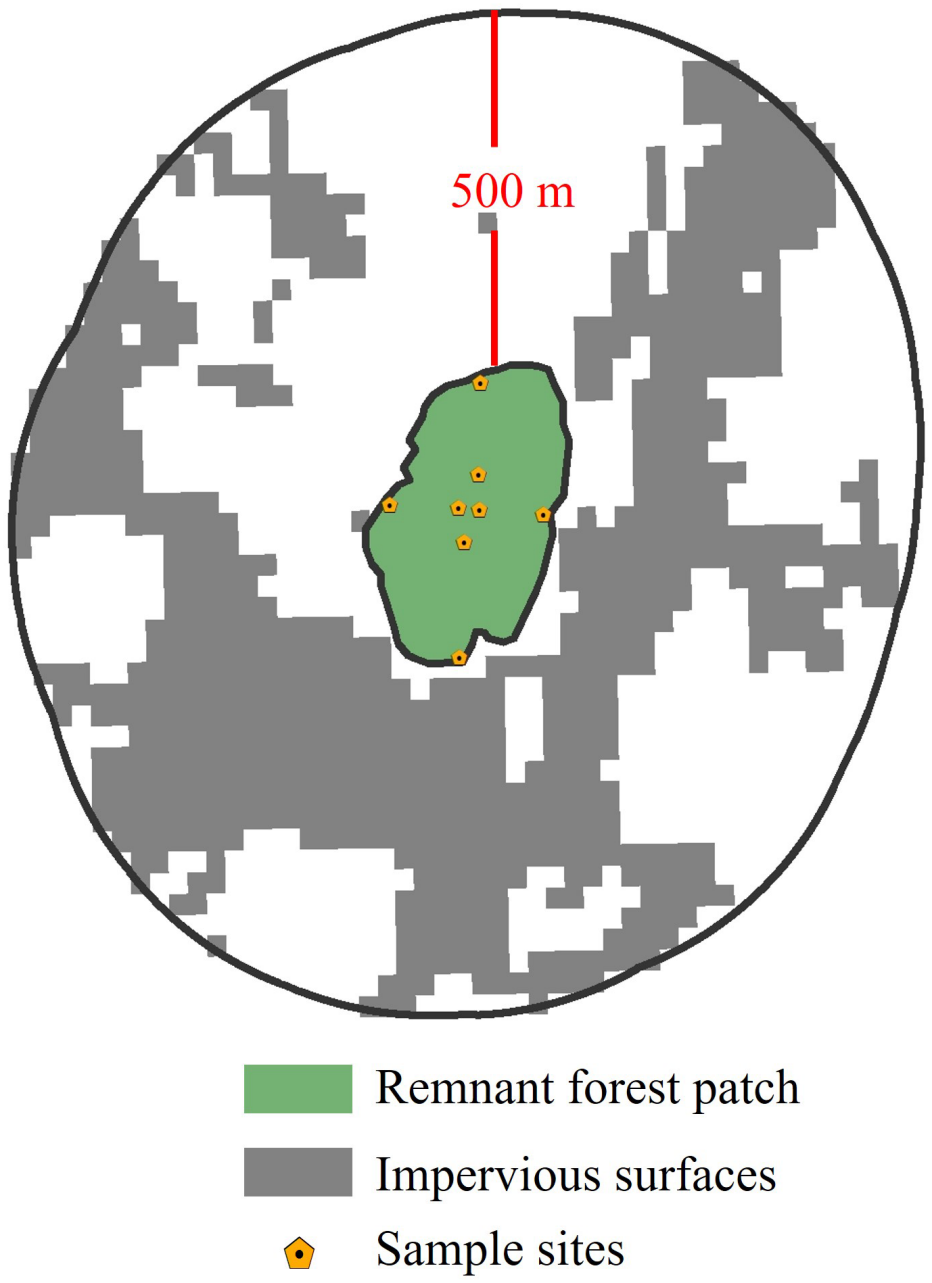
Impervious surfaces within 500 m of the remnant patches. The colour white denotes land cover types other than impervious surfaces.

**FIGURE 2:**
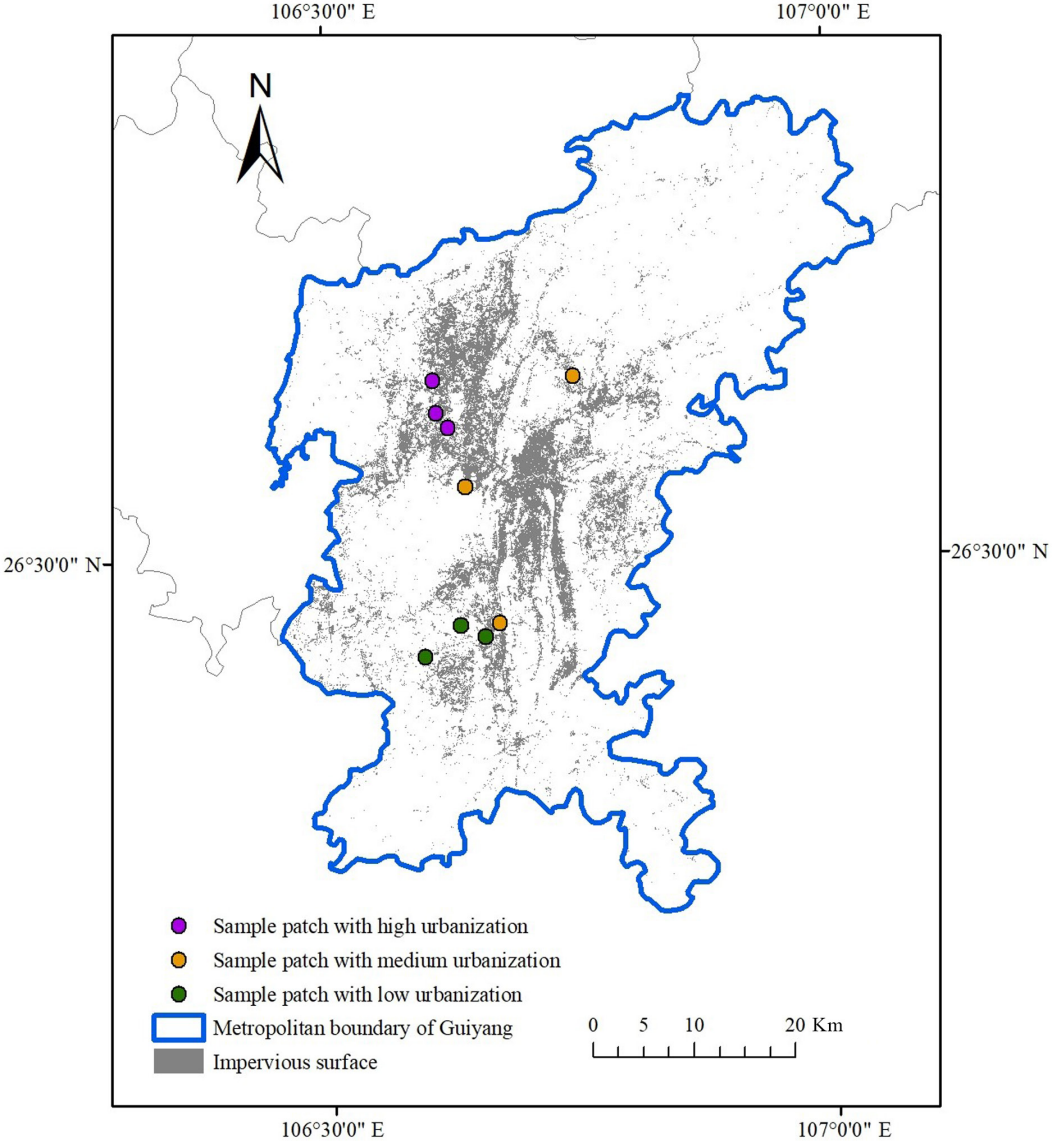
Study area and spatial locations of sample patches with different levels of urbanization.

Urban remnant forest patches in this study are semi‐natural forests that have retained during the process of urbanization and have not been cleared for urban development purposes. Remnant patches were detected in the previous study conducted by Yang et al. (2021). To measure the degree of urbanization in the surrounding landscape, we used the percentage of impervious surfaces within 500 m of the remnant patches (Figure 1) from a land cover survey with a ground resolution of 30 m obtained from Zhang, Liu, et al. (2021). This distance was found to be optimal for establishing the connection between plant diversity of habitat patches and the urban matrix (Vakhlamova et al., 2014). According to Wickham et al. (2010), we sorted the degrees of urbanization by considering the proportion of impervious surfaces in the surrounding area into three groups—low (<20%), moderate (20%–50%), and high levels of urbanization (>50%). After that, we selected three patches each for each level of urbanization. Patches were separated from one another by at least 1 km. Therefore, a total of nine remnant patches were chosen (Figure 2), and their characteristics are available in Appendix A (Table A1). Each sample patch was divided into two areas: the edge area within 25 m of the boundary and the interior area extending from at least 100 m from the boundary to the centre of the patch. In each of these areas, we identified a sample site (20 m × 20 m) at every cardinal point, totalling eight sample sites at each remnant patch. Therefore, the 72 subsample plots were selected from the nine sample patches. More details about the field survey can be obtained from Wang and Yang (2022).

We conducted a field survey from September to December 2021 and recorded the species name and abundance of all the woody plants in the sampling plots. We determined a woody plant species to be a tree or a shrub according to the Flora of Guizhou (Chen, 2004). The species identified as either a small tree or a shrub in the Flora of Guizhou were uniformly identified as a tree in this study. We quantified the abundance of species by counting the number of individual organisms of both tree and shrub species. In cases where it is difficult to distinguish between individuals of certain shrubs, roots are used as the basis of identification. Shrub specimens with interconnected roots are considered as a single individual. In order to portray the overall state of functional traits for each species, we randomly chose five mature and healthy individuals that were exposed to sunlight per tree species from any sample patch. Subsequently, we selected 5–10 intact and healthy leaves from each individual and transported them to the laboratory for further measurements of leaf functional traits.

We measured nine functional traits that reflect the ecological strategies of resource use, dispersal capacity, survivability and competitiveness (Table 1) (Williams et al., 2015; Zhang, Zheng, et al., 2021). Leaf functional trait measurement was conducted following the protocol proposed by Pérez‐Harguindeguy et al. (2013). When leaves were fresh, we measured leaf area with the WINSEEDLE measurement system and leaf thickness with an electronic micrometre. The dry weights of these leaves were measured after oven‐drying at 70°C for nearly 72 h until a constant weight was achieved. The ratio of fresh leaf area to leaf dry weight was calculated as the specific leaf area. The leaf N and C contents were measured by the potassium dichromate‐sulfuric acid oxidation method and indophenol blue colorimetry method, respectively. We collected seed mass and germination rate data from the literature and online resources (see Appendix B, Table B1). When selecting data for seed germination rate, we require that the data be obtained under suitable experimental conditions for seed germination. Therefore, the seed germination rate here refers to the potential germination capacity of plant seeds, without considering the influence of environmental conditions on the germination rate. We collected maximum height data from an online database on flora in China (Chinese Academy of Sciences, 2009).

**TABLE 1:** Nine functional traits and descriptions.

Trait	Description	Unit	Strategy group
Leaf N content	Nitrogen content per dry weight	g/kg	Resource use
Leaf C content	Carbon content per dry weight	g/kg	Resource use
Leaf C/N	The ratio of carbon content and nitrogen content	‐	Resource use
Leaf thickness	The thickness of a fresh leaf	cm	Resource use
Leaf area	The mean area of a single leaf	cm^2^	Resource use
Specific leaf area	The leaf area per dry weight	mm^2^/g	Resource use
Seed germination rate	The ratio of germination number and total number of seed	%	Survivability
Seed mass	The mass per 1000 grains of seed	g	Dispersal, survivability
Maximum height	Plant height at maturity	m	Competitiveness

### 2.3 Categories of species groups

**FIGURE 3:**
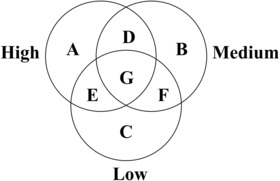
Diagram of categories of species groups. The left, right and bottom circles represent the species that occurred in the remnant patches under high, medium and low levels of urbanization, respectively. A represents the species that only occurred in the patches under high urbanization; B represents the species that only occurred in the patches under medium urbanization; C represents the species that only occurred in the patches under low urbanization; D represents the species that both occurred in the patches under high and medium urbanization; E represents the species that both occurred in the patches under high and low urbanization; F represents the species that both occurred in the patches under medium and low urbanization; G represents the species that simultaneously occurred in the patches under three levels of urbanization.

We classified the recorded species into distinct groups based on their presence‐absence data across various levels of urbanization. The species that only occurred in patches under high urbanization and not under low urbanization were labelled as the ‘urban group’ (A and D in Figure 3), while the species that only appeared in patches under low urbanization but not under high urbanization were identified as the ‘rural group’ (C and F in Figure 3). Those species that existed in patches under both high and low urbanization were assigned to the ‘general group’ (E and G in Figure 3). Additionally, we categorized species that solely appeared in patches under medium levels of urbanization as the ‘middle group’ (B in Figure 3). In this way, our method guarantees that each species is exclusively associated with a single group and is not allocated to multiple groups at the same time.

### 2.4 Phylogeny reconstruction

We downloaded complete chloroplast genome data of recorded species from the National Center for Biotechnology Information (NCBI, https://www.ncbi.nlm.nih.gov/) database. We used sequence data from a congeneric relative when sequence data from the focal species was not available. We compiled and aligned the genetic data using MAFFT v7.477. Then, we reconstructed the phylogeny of aligned sequences using IQ‐TREE v2.0.3 based on the maximum‐likelihood method. The phylogenetic tree files can be found in the Mendeley Data Repository.

### 2.5 Testing for phylogenetic signal

We used Blomberg's *K* as a metric of phylogenetic signal. Blomberg's *K* method proposed by Blomberg et al. (2003) can measure the strength of the phylogenetic signal in continuous functional traits, detect the correlation between functional traits and species evolutionary history, and make comparisons among different traits and phylogenetic trees. Blomberg's *K* is the ratio of phylogenetically correct mean squared error (MSE_0_) divided by the mean square error based on variance–covariance matrix derived from candidate tree (MSE), standardized by the expected MSE_0_/MSE under Brownian motion (Blomberg et al., 2003). The significance of the phylogenetic signal in Blomberg's *K* was tested by comparing the observed evolution of each functional trait to a null model of randomly exchanging trait values across the phylogeny 999 times. To explore the phylogenetic signal in different groups of species, Blomberg's *K* of each category of species mentioned above was calculated, and the significance was also tested.

### 2.6 Analysing the relationship between functional traits and species abundance

We analysed the relationships between functional traits and abundances of each group of species by fitting a linear model using generalized least squares (GLS). Nevertheless, considering the phylogenetic relationship between different species, phylogenetic generalized least squares (PGLS) was also fitted to remove the influence of phylogeny on the functional traits (Blomberg et al., 2012). PGLS is the most commonly used method in phylogenetic comparative studies. It employs a modification of generalized least squares and incorporates knowledge of the phylogenetic relationships to estimate the expected covariance in cross‐species data (Symonds & Blomberg, 2014).

All statistical analyses were conducted in R version 3.5.3 (R Core Team, 2019). Blomberg's *K* was calculated using the *picante* package. GLS and PGLS were all fitted using the *nlme* package.

## 3 RESULTS

**FIGURE 4:**
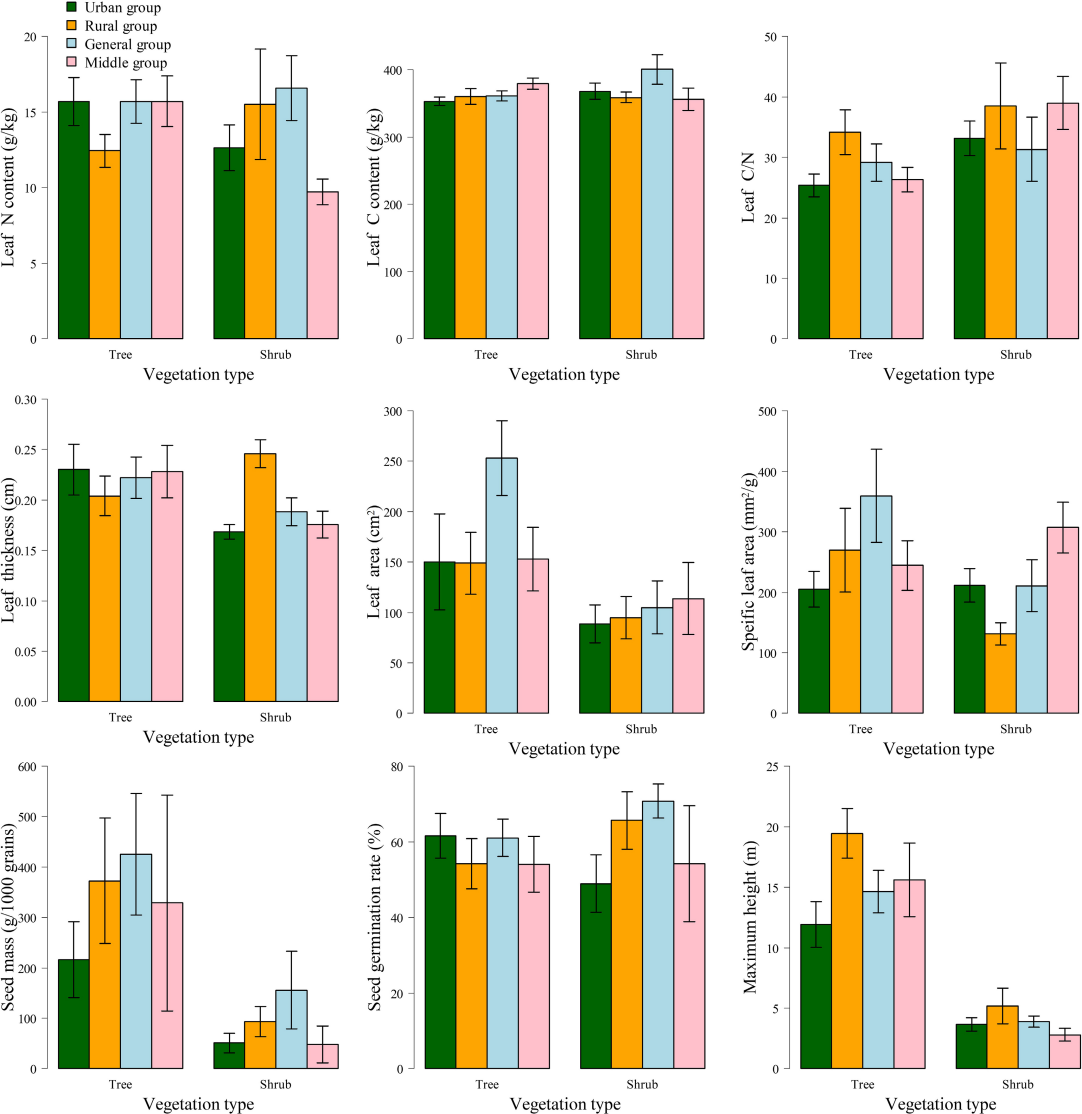
Functional traits of woody plants in different groups. The bar is the mean value, and the arrow is the standard error (95% confidence level).

The functional traits of the four species groups for both tree and shrub species are shown in Figure 4. The mean values of leaf area, specific leaf area and seed mass were higher in the general group of tree species than in the other groups. Additionally, the rural group exhibited higher mean values of leaf C/N and maximum height. Regarding shrub species, the general group had higher mean values of leaf N content, leaf C content, seed mass and seed germination rate compared with the other groups. Similarly, the rural group showed higher mean values of leaf thickness and maximum height.

As shown in Table 2, leaf thickness (*K* = 0.334, *p* = .020) and maximum height (*K* = 0.289, *p* = .024) were detected to show significant phylogenetic signals in the urban group. For the middle group, two traits (leaf C/N and seed mass) had significant phylogenetic signals (*K* = 0.298, *p* = .029; *K* = 2.649, *p* = .004).

**TABLE 2:** Phylogenetic signal of functional traits for tree species based on Blomberg's *K*.

Functional traits	Urban group		Rural group		General group		Middle group	
	*K*	*p*	*K*	*p*	*K*	*p*	*K*	*p*
Leaf N content	0.100	.391	0.077	.669	0.039	.793	0.045	.464
Leaf C content	0.228	.219	0.100	.667	0.005	.998	0.003	.984
Leaf C/N	0.123	.158	0.115	.478	0.105	.905	**0.298**	**.029**
Leaf thickness	**0.334**	**.020**	0.087	.774	0.095	.313	0.017	.655
Leaf area	0.050	.813	0.237	.347	0.109	.248	0.112	.224
Specific leaf area	0.125	.191	0.424	.096	0.364	.230	0.005	.982
Seed germination rate	0.063	.413	0.255	.459	0.155	.121	0.020	.608
Seed mass	0.426	.212	0.155	.551	0.155	.314	**2.649**	**.004**
Maximum height	**0.289**	**.024**	0.291	.142	0.044	.542	0.174	.095

*Note*: Statistical significance at the level of *p* < .05 is indicated by text in bold.

For shrub species (Table 3), the leaf C content (*K* = 1.753, *p* = .003), leaf C/N, leaf thickness (*K* = 0.293, *p* = .030) and seed mass (*K* = 0.288, *p* = .038) of the urban group all had significant phylogenetic signals. In addition, the seed mass of rural species also had a significant phylogenetic signal (*K* = 1.118, *p* = .038). However, we failed to detect phylogenetic signals in other functional traits of shrub species (*p* > .05).

**TABLE 3:** Phylogenetic signal of functional traits for shrub species based on Blomberg's *K*.

Functional traits	Urban group		Rural group		General group		Middle group	
	*K*	*p*	*K*	*p*	*K*	*p*	*K*	*p*
Leaf N content	0.271	.069	0.466	.437	0.253	.160	0.260	.821
Leaf C content	**1.753**	**.003**	0.243	.515	0.037	.889	0.295	.596
Leaf C/N	**0.293**	**.030**	0.178	.659	0.067	.836	0.418	.199
Leaf thickness	**0.224**	**.050**	0.322	.427	0.077	.751	0.301	.887
Leaf area	0.049	.540	0.690	.186	0.259	.263	0.958	.059
Specific leaf area	0.140	.426	0.070	.871	0.398	.193	0.633	.173
Seed germination rate	0.025	.606	0.812	.084	0.044	.967	0.205	.915
Seed mass	**0.288**	**.038**	**1.118**	**.038**	0.695	.068	0.372	.360
Maximum height	0.103	.199	0.793	.120	0.078	.773	0.324	.654

*Note*: Statistical significance at the level of *p* < .05 is indicated by text in bold.

We found that there was a significant correlation between the maximum height trait of tree species and the species abundance of the urban, general, and middle groups, after removing the effects of phylogenetic nonindependence (*p* < .01, see Table 4). Leaf N content (*t* = −8.813, *p* < .001; *t* = 10.191, *p* < .001), leaf C content (*t* = 13.630, *p* < .001; *t* = 15.531, *p* < .001), leaf C/N (*t* = 10.816, *p* < .001; *t* = −3.214, *p* = .009) and maximum height (*t* = −5.064, *p* < .001; *t* = 5.798, *p* = .009) were found to be significantly associated with the species abundance of the general and middle groups. Furthermore, leaf thickness (*t* = −9.775, *p* < .001), specific leaf area (*t* = 11.060, *p* < .001) and seed germination rate (*t* = 13.488, *p* < .001) were also observed to have a significant correlation with the species abundance of the middle group.

**TABLE 4:** Results of generalized least squares (GLS) and phylogenetic generalized least squares (PGLS) for tree species.

Functional traits	Model	Urban group		Rural group		General group		Middle group	
		*t*	*p*	*t*	*p*	*t*	*p*	*t*	*p*
Leaf N content	GLS	−0.041	.968	−1.228	.237	−0.312	.758	1.741	.112
	PGLS	−0.484	.636	−0.907	.378	**−8.813**	**<.001**	**10.191**	**<.001**
Leaf C content	GLS	0.863	.402	−0.955	.354	2.592	.015	1.177	.266
	PGLS	−0.137	.893	0.169	.868	**13.630**	**<.001**	**15.531**	**<.001**
Leaf C/N	GLS	0.083	.935	1.358	.193	0.218	.829	−1.621	.136
	PGLS	0.409	.688	−0.955	.354	**10.816**	**<.001**	**−3.214**	**.009**
Leaf thickness	GLS	−0.023	.982	0.044	.966	0.547	.589	0.553	.592
	PGLS	−0.709	.489	−0.401	.694	2.214	.036	**−9.775**	**<.001**
Leaf area	GLS	0.038	.971	0.236	.817	0.863	.396	−0.029	.977
	PGLS	−0.383	.707	0.400	.694	**3.593**	**.001**	1.119	.289
Specific leaf area	GLS	−1.500	.154	−0.140	.890	−0.520	.608	1.417	.187
	PGLS	−1.408	.180	−0.367	.718	−1.869	.073	**−11.060**	**<.001**
Seed germination rate	GLS	0.805	.434	−1.562	.138	−1.349	.189	−0.530	.609
	PGLS	0.722	.481	−0.943	.360	−0.559	.581	**−13.488**	**<.001**
Seed mass	GLS	−0.877	.394	−0.498	.625	−0.550	.587	−0.872	.403
	PGLS	−0.764	.457	−0.802	.435	−1.897	.069	−0.172	.867
Maximum height	GLS	2.519	.024	−0.887	.388	0.408	.686	−0.703	.498
	PGLS	**4.025**	**.001**	−0.683	.505	**−5.064**	**<.001**	**5.798**	**<.001**

*Note*: Statistical significance at the level of *p* < .01 is indicated by text in bold.

**FIGURE 5:**
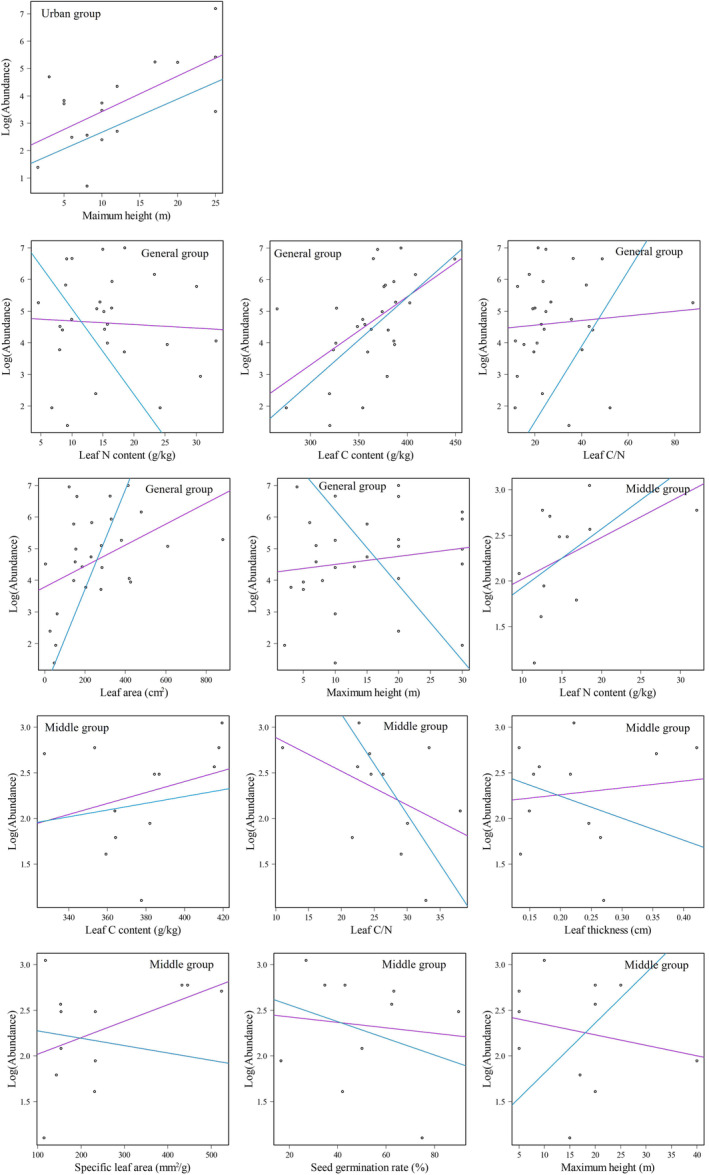
Significant relationship (*p* < .01) between functional traits and species abundance for tree species based on generalized least squares (purple lines) and phylogenetic generalized least squares (blue lines). Circles represent scatter plots showing the relationship between predictor and response variables.

We observed that the maximum height had a positive association with the tree species abundance of the urban and middle groups and a negative association with the general group after removing the phylogenetic signal (Figure 5). The leaf N content and leaf C/N trait relationship with species abundance was reversed for the middle and general groups. Furthermore, the seed germination rate and specific leaf area showed a negative association with species abundance in the middle group.

No significant relationship was found between the abundance of shrub species in the urban, rural and middle groups and any of the tested traits (*p* > .01, Table 5). In contrast, the abundance of general shrub species was significantly correlated with three traits: leaf nitrogen content (*t* = 3.643, *p* = .003), leaf thickness (*t* = 4.248, *p* = .001) and leaf area (*t* = 3.249, *p* = .007) after removing phylogenetic signals.

**TABLE 5:** Results of generalized least squares (GLS) and phylogenetic generalized least squares (PGLS) for shrub species.

Functional traits	Model	Urban group		Rural group		General group		Middle group	
		*t*	*p*	*t*	*p*	*t*	*p*	*t*	*p*
Leaf N content	GLS	0.121	.906	−1.124	.287	−0.775	.454	2.030	.089
	PGLS	−0.199	.846	−0.746	.473	**3.643**	**.003**	2.800	.031
Leaf C content	GLS	−0.528	.607	1.404	.191	0.104	.919	2.107	.080
	PGLS	−0.341	.739	−0.777	.455	−0.539	.600	2.455	.049
Leaf C/N	GLS	−0.661	.521	2.777	.020	0.682	.508	−0.626	.554
	PGLS	0.546	.595	−0.272	.791	−2.207	.048	−1.379	.217
Leaf thickness	GLS	0.383	.708	0.565	.584	1.049	.315	1.463	.194
	PGLS	−0.579	.534	2.145	.058	**4.248**	**.001**	−0.626	.554
Leaf area	GLS	0.852	.411	0.737	.478	−0.644	.532	−0.191	.855
	PGLS	1.867	.087	−0.039	.970	**3.249**	**.007**	−0.573	.588
Specific leaf area	GLS	0.686	.506	−1.249	.240	−0.336	.743	−1.335	.230
	PGLS	−1.103	.292	−3.059	.012	−0.228	.824	−3.148	.020
Seed germination rate	GLS	0.276	.788	0.425	.680	−1.133	.279	0.431	.696
	PGLS	−0.069	.946	0.517	.617	−2.059	.062	0.894	.438
Seed mass	GLS	−2.151	.053	−0.585	.572	−1.133	.279	−0.102	.922
	PGLS	−0.719	.486	−0.398	.699	−2.059	.062	−0.144	.890
Maximum height	GLS	−0.498	.627	−0.928	.375	−0.224	.827	−2.257	.065
	PGLS	−2.885	.014	−1.184	.264	1.699	.115	−2.018	.090

*Note*: Statistical significance at the level of *p* < .01 is indicated by text in bold.

**FIGURE 6:**
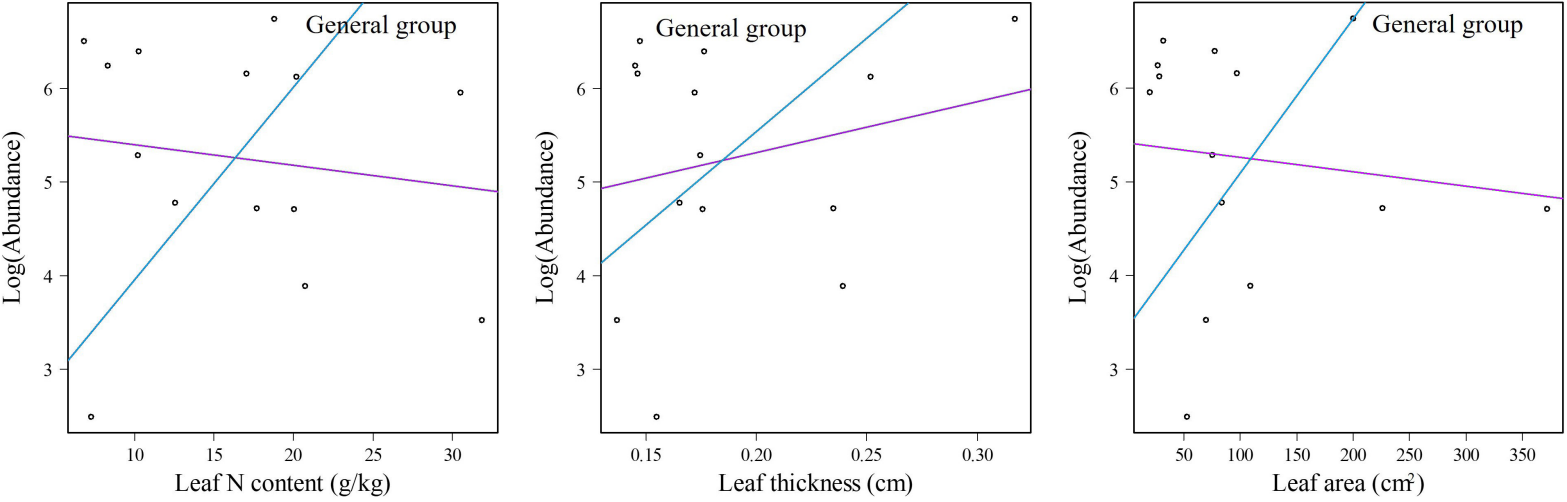
Significant relationship (*p* < .01) between functional traits and species abundance for shrub species based on generalized least squares (purple lines) and phylogenetic generalized least squares (blue lines). Circles represent scatter plots showing the relationship between predictor and response variables.

As shown in Figure 6, leaf N content, leaf thickness and leaf area of shrub species were all positively associated with the abundance of shrub species in the general group.

## 4 DISCUSSION

Our results provide evidence that high levels of urbanization strengthen the phylogenetic signal of functional traits for woody plants in remnant forests. We detected significant phylogenetic signals in more functional traits of the urban group. Urbanization generates more stressful environments, such as heat islands, drought and soil compaction (Devigne et al., 2016; Hirsch et al., 2022; Tian et al., 2021). More stressful conditions may result in the greater presentation of phylogenetic signals in functional traits (Cavender‐Bares et al., 2006). Another study also revealed evidence that phylogenetic constraints are stronger in stressful than benign competitive contexts (Burns & Strauss, 2012). Biological competition allows distantly related species to coexist (Gerhold et al., 2011). Blomberg's *K* of leaf C content in urban shrub species was greater than one, which indicated that this trait was influenced highly by phylogeny. This may be explained by the particular light conditions in urban areas, which are not a determining factor for carbon accumulation in plant leaves (Ebbensgaard, 2015).

There was no detection of a phylogenetic signal of any functional traits for the general group. This pattern can be explained by two aspects. One explanation for the low levels of phylogenetic signals is more convergent adaptation for species in the general group. General species are distributed widely and have evolved separately at different sites and assembled in a local area (Liu et al., 2022). They are often distantly related species but have similar functional traits (Ackerly, 2009). For example, general species often show the ecological strategy of fast and highly efficient resource acquisition (e.g. high specific leaf area) and strong survivability (e.g. high seed mass) (Diaz et al., 2016). Therefore, they can adapt to a variety of environmental conditions. An alternative explanation is that to accommodate changing environments, the functional traits of general species vary along environmental gradients, suggesting a high lability of evolutionary traits rather than conservatism (Ndiribe et al., 2014).

Urbanization has been found to promote the growth of taller tree species, whereas its effect on the height of shrub species remains elusive. Removing the phylogenetic signal revealed that maximum tree height in the urban group was positively linked to abundance, but this was not the case for shrub species. Numerous prior researches discovered that urbanization benefits taller plant species (Williams et al., 2015). Maximum plant height reflects their competitive ability for resources and growth potential in stressful environments (Nock et al., 2013). Trees respond positively to urbanization due to their competitive advantage in hostile environments and longer lifespan (Duncan et al., 2011; Fischer et al., 2013; Thompson & McCarthy, 2008). As a result, there is a higher probability of tall trees being able to survive and persist in remnant forests during urbanization. Conversely, shrub species experience less sunlight competition in the understory and are readily removed by disturbance but easily regenerate (Körner, 2012). Therefore, urbanization has a relatively small impact on the maximum height of shrubs.

There was a significant correlation between leaf area and the abundance of the general group's species. A larger leaf area can enhance a plant's photosynthetic efficiency and water use efficiency (Brodribb et al., 2020; Mantuano et al., 2021). Plant species with larger leaf area facilitates higher rates of photosynthesis, enabling them to convert more sunlight and carbon dioxide into energy. Additionally, larger leaves can provide a competitive advantage by capturing more resources such as water, nutrients and light than smaller‐leaved species. However, for the tree and shrub species, the relationship between species abundance and leaf N content and were opposite. Tree species had a declining leaf N content due to long life and slow growth (Mu & Chen, 2021), while shrubs had higher leaf N content due to their fast growth strategy (Donovan et al., 2011; Zhang, Zheng, et al., 2021). Tree species had a positive correlation between leaf C/N and abundance due to enhanced metabolic activity and growth rate (Sheng et al., 2021; Zhang et al., 2013).

Our findings indicate that the correlation between tree species abundance and four functional traits varied for the middle and general groups, with the former demonstrating a preference for high resource acquisition strategies (such as higher leaf N, lower leaf C/N and lower leaf thickness, although with a higher maximum height). This is in contrast to a high‐resource conservation approach. The moderate urbanization scenario favoured the dominance of fast‐growing and early‐arriving species through medium disturbances (Guler, 2020; Zhang, Zheng, et al., 2021). Additionally, the increased maximum height of middle tree species can be attributed to their ability to thrive in urban environments (Williams et al., 2015). Urban environmental conditions can be more favourable for taller plant species because they have larger photosynthetic surface areas, which allow them to grow and reproduce more efficiently in these environments. Additionally, taller plants can more effectively compete for limited resources such as sunlight and nutrients.

The changes in functional traits of remnant vegetation in urban areas differ depending on whether phylogenetic signals are removed. Our findings demonstrate that when phylogenies were not removed, no noteworthy associations between species abundance and functional traits were identified. However, after phylogenetic signals were removed, 13 and 3 significant relationships were observed for tree and shrub species, respectively. It is critical to establish the credibility of the independence assumption in the models used to explicate the link between functional traits and species abundance. Therefore, a combination of functional trait and phylogenetic information may aid in assessing the ecological impact of urbanization on remnant forest ecosystems.

## 5 CONCLUSION

Plant functional traits and phylogenies can play a vital role in exploring remnant vegetation filtering in urban environments. Most previous studies only focused on taxonomic and functional filtering while ignoring the effect of phylogenies. In this study, we found significant phylogenetic signals in many plant functional traits, especially for urban groups. Moreover, the phylogenies significantly affected our ability to detect dominant functional traits for different groups of woody plant species. These results have critical implications for understanding plant assembly in urban remnant forests, especially those that use phylogenetic analysis of functional traits and community structure to infer mechanisms of species coexistence. Studies combining functional traits with phylogenetic analysis of urban remnant vegetation can provide new insight into the mechanism of plant community function and productivity. Future studies can further explore the effects of urbanization on the functional and phylogenetic traits of remnant vegetation across more temporal and spatial scales.

## AUTHOR CONTRIBUTIONS

**Jingyi Yang:** Conceptualization (equal); data curation (equal); formal analysis (equal); funding acquisition (equal); methodology (equal); project administration (equal); resources (equal); software (equal); supervision (equal); validation (equal); visualization (equal); writing – original draft (equal); writing – review and editing (equal). **Zijin Wang:** Data curation (equal); investigation (equal); methodology (equal); validation (equal); visualization (equal); writing – review and editing (equal). **Ying Pan:** Data curation (equal); investigation (equal); methodology (equal); validation (equal); writing – review and editing (equal). **Yanjun Zheng:** Data curation (equal); investigation (equal); methodology (equal); visualization (equal); writing – review and editing (equal).

## FUNDING INFORMATION

This research was funded by the Guizhou Science and Technology Department under Grant (QKHLHZ[2016]7447)

## CONFLICT OF INTEREST STATEMENT

The authors declare no conflicts of interest.

## Data Availability

The data for this study are available via the Mendeley Data Repository. https://doi.org/10.17632/z5g82bjm95.1.

## APPENDIX A

**TABLE A1:** Characteristics of the sample patches.

ID	Levels of urbanization	Percentage of impervious surface(%)	Geographical location
1	High	74.7	26°39′35.62″ N, 106°36′26.19″ E
3		75.6	26°37′52.06″ N, 106°36′35.68″ E
4		56.8	26°37′5.46″ N, 106°37′19.64″ E
2	Medium	37.3	26°39′44.97″ N, 106°44′50.8″ E
5		21.7	26°33′53.89″ N, 106°38′15.42″ E
7		48.6	26°26′36.97″ N, 106°40′13.56″ E
6	Low	3.5	26°26′30.63″ N, 106°37′54.52″ E
8		19.1	26°25′52.98″ N, 106°39′18.94″ E
9		14.1	26°24′51.44″ N, 106°35′45.18″ E

## APPENDIX B

**TABLE B1:** The reference sources of seed trait data.

Name	Source
*Alangium chinense*	Yu Bin, Wang Cheng. Seeding‐raising experiments of Alangium platanifolium. *Protection Forest Science and Technology*, 2015, (10), 41–43.
*Robinia pseudoacacia*	https://zhidao.baidu.com/question/205589573143906285
*Trachycarpus fortunei*	https://www.cmeii.com/huapuriji/88106.html
*Kalopanax septemlobus*	Han Youzhi. Study on seed removing dormancy and germination seedling raising of *Kalopauax septemlobus* in Northeast China. *Liaoning Academy of Forestry*, 2020, 45(2), 1–5.
*Ilex corallina*	https://www.taodocs.com/p‐338155629.html
*Aralia chinensis*	She Ping, Yu Zhijia, Ma Jie, Jia Baoguang, Wang Zhengan. Effects of accelerating germination and covering on seed germination and growth of *Aralia chinensis*. *Guyuan Branch of Ningxia Academy of Agriculture and Forestry Sciences*, 2021, 39(2), 244–250.
*Toricellia tiliifolia*	Hua Qi. Cutting propagation technology of *Toricellia angulata* var. *intermedia*. *Chinese Agricultural Science Bulletin*, 2011, 27(28), 258–262.
*Itea yunnanensis*	Bi Bo. Preliminary report on cutting propagation experiment of *Rattus yunnanensis*. *Guangxi Forestry Science*, 2010, 39(1).
*Ilex chinensis*	https://wenku.baidu.com/view/80c3a08ecfc789eb162dc862.html
*Elaeocarpus decipiens*	https://www.cmeii.com/yanghuajiqiao/85793.html
*Quercus fabri*	Liu Renlin, Zhu Heng, Li Jiao. Dynamic laws six nutrient ingredients in fruits of quercus fabric. *Nonwood Forest Research*, 2009, 27(4), 7–11.
*Rhamnella martinii*	https://jz.docin.com/p‐985247140.html
*Koelreuteria paniculata*	https://www.cmeii.com/baiwenbaida/68690.html
*Broussonetia papyifera*	https://www.cmeii.com/huashibaike/70321.html
*Cornus walteri* Wanger	Shaoping Chen. Characteristics and seedling raising techniques of *Betula luminifera*. *Modern Agricultural Science and Technology*, 2019, (14), 144–144+146.
*Prunus laurocerasus*	Xiaoyao Li. Study on seed quality identification and seeding and seedling raising techniques of *Cinnamomum camphora*. *Modern Agricultural Science and Technology*, 2015, (12).
*Cupressus funebris*	Luo Chengong. A study of the seed vigor of *Cupressus funebris* Endl. *Journal of Sichuan Forestry Science and Technology*, 1992, (2).
*Meliosma oldhamii*	https://wenku.baidu.com/view/e1a8f4023186bceb18e8bb47
*Rhus punjabensis*	Xie Shuming, et al. Seed quality and seedling cultivation techniques of *Populus rubra*. *Journal of Sichuan Forestry Science and Technology*, 1993, (1).
*Zanthoxylum bungeanum*	https://www.52shihu.com/plan/432510622432512019/
*Platycarya longipes*	https://jz.docin.com/p‐745828661.html
*Morus australis*	https://www.52shihu.com/plan/394100622394102019/
*Populus canadensis*	https://jz.docin.com/p‐745828661.html
*Castanea mollissima*	https://wenku.baidu.com/view/8d3d100a3968011ca3009141.html
*Swida macrophylla*	Wang Minghuai. Study on the fruit characteristics and yield of *Cornus wilsoniana*. *Forestry and Environmental Science*, 2017, 33(3), 24–28.
*Photinia davidsoniae*	Ou Bing. Experimental study on field seedling raising of *Photinia pallidum*. *Forest By‐Product and Speciality in China*, 2009, (3).
*Quercus acutissima*	http://www.yuanlin365.com/yuanyi/203951.shtml
*Pinus massoniana*	Duan Qiong. Variation law of 1000‐grain weight of *Pinus massoniana* and its application. *Journal of Sichuan Forestry Science and Technology*, 2002, (2).
*Litsea pungens*	Wu Canglong. Study on selection of excellent individual plant of *Litsea cubeba*. *Private Science and Technology*, 2015, (1).
*Choerospondias axillaris*	Wang Ling. Characteristics and seed seedling cultivation and afforestation techniques of south sour jujube in the southern anhui mountain area. *Modern Agricultural Science and Technology*, 2019, 746(12), 127–135.
*Ligustrum lucidum*	https://www.cmeii.com/huashibaike/78201.html
*Platycladus orientalis*	https://www.mucaohui.com/FAQ/26701.html
*Paulownia fortunei*	https://www.cmeii.com/yanghuajiqiao/78112.html
*Eriobotrya japonica*	https://www.xuexila.com/zhishi/zhiwu/3850392.html
*Celtis sinensis*	https://www.cmeii.com/huashibaike/76409.html
*Toxicodendron vernicifluum*	https://www.cmeii.com/huapuriji/88583.html
*Schoepfia jasminodora*	Jiang Guozhi. Preliminary study on seeding and seedling raising techniques and seedling growth regularity of *Picea wilsoniana*. *Anhui Forestry Science and Technology*, 2013, 39(3), 78–79.
*Catalpa bungei*	Guo Congjian. Relationship between seed picking time and seed quality of trees. *Journal of Henan Agricultural University*, 1990, (2).
*Albizia kalkora*	https://www.cmeii.com/news/68347.html
*Albizia kalkora*	https://www.docin.com/p‐1006274405.html
*Celtis julianae*	Wu Xiaoxian. Study on the seed biological characteristics of *Celtis julianae*. *Journal of Forestry Engineering*, 2008, (5).
*Cudrania tricuspidata*	Tao Guanglin. Investigation of Zhemu fruit. *Chinese Wild Plant Resources*, 2005, (2).
*Evodia rutaecarpa*	Liu Shanshan. Yin Yuanyuan. Yan Lihua. Investigation on resources of medicinal plant of Euodiae Fructus. *Chinese Journal of Information on Traditional Chinese Medicine*, 2016, 23(9), 5–9.
*Firmiana simplex*	https://www.docin.com/p‐809833526.html
*Camptotheca acuminata*	Characteristics of *Camptotheca acuminata* and seedling raising and afforestation techniques. *Modern Agricultural Science and Technology*, 2020, (4).
*Eurya nitida*	Pan Jian. The experimental analysis on the techniques of propagation for *Eurya nitida*. *Journal of Nanjing Forestry University (Natural Sciences Edition)*, 2005, (6).
*Eurya loquaiana*	https://www.52shihu.com/plan/40831062240S12019/
*Cladrastis platycarpa*	https://www.cmeii.com/yanghuajiqiao/88324.html
*Toona sinensis*	Zhang Li‐Yun. Seed characters and seedling growth of different provenances of *Toona sinensis* Roem. *Journal of Anhui Agricultural Sciences*, 2017, 45(8).
*Lindera communis*	Kang Yong‐Wu. Study on the variation of height and ground diameter of one‐year‐old *Lindera* increment. *Subtropical Plant Science*, 2013, 42(3).
*Cinnamomum glanduliferum*	https://www.cmeii.com/yanghuajiqiao/76608.html
*Populus adenopoda*	Tang Qiang. Research progress of *Populus euphratica*. *Hunan Forestry Science & Technology*, 2011, 38(4).
*Ficus tinctoria*	Zhang Jintang. Study on seed biological characteristics and seedling growth characteristics of four species of *Ficus*. *Seed*, 2018, 37(9).
*Rhus chinensis*	https://www.cmeii.com/huapuriji/87665.html
*Myrica rubra*	https://www.cmeii.com/huapuriji/65850.html
*Populus simonii*	http://www.hm160.cn/html/9013978.htm
*Crataegus cuneata*	https://www.cmeii.com/news/65565.html
*Diospyros kaki*	https://www.cmeii.com/news/66410.html
*Nothopanax davidii*	https://jz.docin.com/p‐745828661.html
*Ficus heteromorpha*	https://www.cmeii.com/huapuriji/83743.html
*Cerasus pseudocerasus*	https://www.cmeii.com/huashibaike/65075.html
*Citrus maxima*	https://www.cmeii.com/huapuriji/65248.html
*Corylus heterophylla*	Wang Lujun. Preliminary cultivation experiment report on introduced *Corylus heterophylla* var. *sutchuenensis*. *Anhui Forestry Science and Technology*, 2014, (4), 25–27.
*Ulmus pumila*	https://www.cmeii.com/huashibaike/81519.html
*Carpinus pubescens*	Wen Yan. Seed‐setting characteristic of *Carpinus pubescens* population in Karst Area. *Guizhou Agricultural Sciences*, 2010, 38(3).
*Cinnamomum glanduliferum*	Liao Wanbing. Study on growth and development of *Cinnamomum glanduliferum* annual seedling. *Journal of Mountain Agriculture and Biology*, 2004, (3).
*Xylosma racemosum*	https://www.zhiwuwang.com/news/20268.html
*Hovenia acerba*	Li Ying. A study on the seed dormancy mechanism and ways of dormancy breaking in *Hovenia acerba* Lindl. *Journal of Nanjing Forestry University (Natural Sciences Edition)*, 2014, 38(2).
*Euonymus nitidus*	Men Siyuan. Physiological response to cold stress and evaluation of cold resistance for five species of *Euonymus* Linn. *Acta Botanica Boreali‐Occidentalia Sinica*, 2020, 40(4).
*Celtis biondii* Pamp	Xia Shangguang. Preliminary report on introduction and seedling raising experiment and seedling growth law of Coral Park and *Rhododendron purpurea*. *Anhui Agricultural Science Bulletin*, 2013, 19(12).
*Citrus reticulata*	Zhu Shiping. Seed traits and seedling performances of different types of *Citrus* rootstock. *Scientia Agricultura Sinica*, 2020, 53(3).
*Zanthoxylum esquirolii*	https://www.52shihu.com/plan/432510622432512019/
*Pittosporum tobira*	https://www.cmeii.com/huapuriji/82688.html
*Picrasma quassioides*	https://www.52shihu.com/plan/433600622433602019/
*Ginkgo biloba*	https://www.docin.com/p‐46122651.html
*Fatsia japonica*	Zhao Xuejiao. Relationships between population and habitat characteristics and reproduction on artificial population of the *Fatsia japonica*. Sichuan: Sichuan Agricultural University, 2016.
*Smilax china*	Guo Yongqing. Studies on introduction and propagation of wild *Smilax china* in Lao shan. Shandong: Qingdao Agricultural University, 2008.
*Acanthopanax trifoliatus*	Xiao Juan. On seed dormancy mechanism of *Acanthopanax trifoliatus* (L.) Merr. *Journal of China West Normal University (Natural Science)*, 2014, 35(3), 201–206.
*Sarcococca ruscifolia*	Long Jiao. Introduction cultivation of *Sarcococca ruscifolia* Stapf and its application in urban landscape. *Guizhou Agricultural Sciences*, 2007, 35(6), 46–47, 51.
*Rhamnus leptophylla*	https://www.docin.com/p‐46122651.html
*Prinsepia utilis*	Zhang Wangjun, Yang Xiaopeng. Development value and cultivation techniques of *Prinsepia utelis* Royle. *Shanxi Agricultural Sciences*, 2008, 36(2), 82–83.
*Clerodendrum chinense*	Ruan Xiaoying. Cultivation and application of *Verbena officinalis* L. *Flower Plant & Penjing*, 2019, (9).
*Rubus setchuenensis*	Jin Wei. Zheng Shengzhi. Tissue culture and rapid propagation of Rubus setchenensis. *Plant physiology Communication*, 1991(4), 290–291.
*Ficus tikoua*	https://wenku.baidu.com/view/db3ea32b3169a4517723a367.html
*Indigofera amblyantha*	Li Chaofeng. Study on characteristics of hard seeds and germination of *Indigofera amblyantha* Craib. *Journal of Grassland and Forage Science*, 2007(12), 8–10.
*Toddalia asiatica*	http://www.cnki.com.cn/Article/CJFDTOTAL‐ZNYK202003011.htm
*Rubus biflorus*	Li Weilin, Jiang Zhenjun, Jiang Xuyin. Study on development and utilization of *Rubus parvifolius* resources. *Soil and Water Conservation in China*, 1994, (6), 37–39.
*Sageretia henryi*	Qian Lianfang, Li Zhangju, Qian Yongtao, Gao Hong. Reproduction experiment of four species of *Bromus*. *Journal of Zhejiang Forestry University*, 1995(4), 374–379.
*Berchemia polyphylla*	https://jz.docin.com/p‐985247140.html
*Rhamnus esquirolii*	https://jz.docin.com/p‐985247140.html
*Pittosporum illicioides*	https://www.cmeii.com/huapuriji/82688.html
*Berberis julianae*	https://www.cmeii.com/huapuriji/75568.html
*Periploca forrestii*	Zhang Ming, Yang Xia, Yang Guozheng, et al. Tissue culture and plantlet regeneration of *Periploca forrestii* Schltr. *Plant Physiology Communications*, 2007, 43(4), 752.
*Elaeagnus pungens*	Fu Chao, Liu Qian, Qiu Fengying, et al. Study on fruit quality and seed germination of *Elaeagnus conferta*. *Southern Forestry Science*, 2018, 46(3), 13–15, 19.
*Zanthoxylum scandens*	https://www.52shihu.com/plan/432510622432512019/
*Pyracantha fortuneana*	http://www.hm160.cn/20124/84526826.htm
*Viburnum chinshanense*	Yang Chunyu, Wu Xiaoli, Yuan Maoqin, et al. Effect of different temperatures on germination of *Viburnum chinshanense* seed. *Seeds*, 2013, 32(10), 43–45.
*Hypericum monogynum*	Zhang Zushuai. Researches on fruit, seed morphology and germination characteristic of *Hypericum chinensis*. *Shanxi Forestry Science and Technology*, 2009, 38(1), 26–28.
*Stachyurus chinensis*	Zhu Shiping. Wang Fusheng. Chen Jiao. Seed Traits and Seedling Performances of Different Types of Citrus Rootstock. Agricultural Sciences in China, 2020, 53(03), 585–599.
*Celastrus angulatus*	Meng Shiyuan, Lu Guiyun, Zhang Mingzhong, et al. Physiological response to cold stress and evaluation of cold resistance for five species of *Euonymus* Linn. *Acta Botanica Sinica*, 2020, 40(4), 624–634.
*Lonicera fragrantissima*	https://www.cmeii.com/baiwenbaida/96042.html
*Chimonanthus praecox*	https://www.cmeii.com/baiwenbaida/70696.html
*Brandisia hancei*	Xu Ying, Chen Xuewei, Ren Yongquan, et al. Advances of research on *Brandisia hancei*. *Journal of Kaili University*, 2015, 33(6), 87–89.
*Sageretia lucida*	Qian Lianfang, Li Zhangju, Qian Yongtao, Gao Hong. Reproduction experiment of four species of *Bromus*. *Journal of Zhejiang Forestry University*, 1995(4), 374–379.
*Serissa japonica*	Shu Yinglan. VI. Tissue culture of in the snow. *Plant Physiology Communications*, 1984(3), 45–46.
*Nothapodytes pittosporoides*	https://wenku.baidu.com/view/e1a8f4023186bceb18e8bb47
*Coriaria nepalensis*	https://www.cmeii.com/yuanyishifenxiang/96956.html
*Elaeagnus glabra*	Fu Chao, Liu Qian, Qiu Fengying, et al. Study on fruit quality and seed germination of *Elaeagnus conferta*. *Southern Forestry Science*, 2018, 46(3), 13–15, 19.
*Celastrus rosthornianus*	Li Yinhua, Guo Weizhen, Xing Cunwang, et al. Sowing and seedling raising technology and ornamental application of *Celartrus scandens*. *Anhui Agricultural Sciences*, 2011, 39(7), 4078–4079, 4082.
*Nandina domestica*	https://www.cmeii.com/baiwenbaida/78708.html
*Helwingia chinensis*	https://www.cmeii.com/news/78424.html
*Viburnum propinquum*	Zhang Lin. Collection and utilization of some germplasm resources of Viburnum. Shandong Agricultural University, 2007.
*Lindera glauca*	Qi. Analysis on the growth and development law and genetic diversity of *Zanthoxylum piperitum*. Beijing: Beijing Forestry University, 2015.
*Rubus corchorifolius*	Jin Wei. Zheng Shengzhi. Tissue Culture and Rapid Propagation of Rubus setchenensis. *Plant physiology Communication*, 1991(4), 290–291.
*Mallotus repandus*	https://www.cmeii.com/news/84070.html
*Debregeasia orientalis*	Wang Guoying. Germination characteristics and introduction and cultivation techniques of large nettle seeds. *Journal of Forestry Engineering*, 2006(4), 74–75.
*Glochidion puberum*	https://www.cmeii.com/huapuriji/90409.html
*Dalbergia hancei*	https://www.cmeii.com/yanghuajiqiao/72769.html
*Myrsine africana*	Ping, Zhang Xuexing, Chen Haiyun, et al. The sowing and seedling technology of *Myrsine africana*. *Shaanxi Forestry Science and Technology*, 2018, 46(1), 100–102.
*Rubus parkeri*	https://www.cmeii.com/huapuriji/79481.html
*Cotoneaster franchetii*	https://max.book118.com/html/2017/0724/124036525.shtm
*Rosa cymosa*	https://www.cmeii.com/huapuriji/79481.html
*Ohwia caudata*	https://www.cmeii.com/huapuriji/84610.html
*Ligustrum sinense*	https://zhidao.baidu.com/question/142019678664443845.html
*Cotoneaster hissaricus*	Technical regulations for seedling cultivation of *Cotoneaster*: DB37/T3001‐2017. 2017.
*Zanthoxylum dissitum*	Fei Mingliang. Study on the conditions of seed dormancy and germination of *Zanthoxylum* shell. Hunan: Central South University of Forestry and Technology, 2015.
*Rosa multiflora*	https://www.cmeii.com/huapuriji/79481.html
*Rhamnus heterophylla*	https://jz.docin.com/p‐985247140.html
*Viburnum foetidum*	Zhang Lin. Collection and utilization of some germplasm resources of Viburnum. Shandong Agricultural University,2007.
*Zanthoxylum armatum*	Cai Qinyue. Fu Benning. XU Danping. Effect of different harvesting periods on the quality of Zanthoxylum armatum seeds. *China Oils and Fats*, 2019,44(2), 81–85.
*Ardisia japonica*	Xu Yonghong, Zhou Zhenqi, Peng Jianjian, et al. Study on seed germination characteristics of the rare and endangered plant of *Ardisia violacea*. *Green Science and Technology*, 2021, 23(9), 66–68.
